# Prevention of influenza

**DOI:** 10.1590/S1516-31802009000400012

**Published:** 2009-12-07

There are several Cochrane reviews of the relevant evidence relating to different aspects of the prevention and treatment of influenza. Two abstracts on this topic are presented below. The full texts of these reviews are available free of charge from: http://cochrane.bvsalud.org/portal/php/index.php?lang=pt.

## 1. Interventions for the interruption or reduction of the spread of respiratory viruses^1^

ABSTRACT

BACKGROUND: Although respiratory viruses usually only cause minor disease, they can cause epidemics. Approximately 10% to 15% of people worldwide contract influenza annually, with attack rates as high as 50% during major epidemics. Global pandemic viral infections have been devastating because of their wide spread. In 2003 the severe acute respiratory syndrome (SARS) epidemic affected ~8,000 people, killed 780, and caused an enormous social and economic crisis. A new avian influenza pandemic caused by the H5N1 strain might be more catastrophic. Viral epidemics or pandemics such as of influenza or severe SARS pose a significant threat. Antiviral drugs and vaccination may not be adequate to prevent catastrophe in such an event.

OBJECTIVES: To systematically review the evidence of effectiveness of interventions to interrupt or reduce the spread of respiratory viruses (excluding vaccines and antiviral drugs, which have been previously reviewed).

SEARCH STRATEGY: We searched the Cochrane Central Register of Controlled Trials (CENTRAL) (The Cochrane Library 2006, issue 4); MEDLINE (Medical Literature Analysis and Retrieval System, 1966 to November 2006); OLDMEDLINE (Older Medical Literature, 1950 to 1965); EMBASE (Excerpta Medica Databases, 1990 to November 2006); and CINAHL (Cumulative Index to Nursing and Allied Health Literature, 1982 to November 2006).

SELECTION CRITERIA: We scanned 2300 titles, excluded 2162 and retrieved the full papers of 138 trials, including 49 papers of 51 studies. The quality of three randomised controlled trials (RCTs) was poor; as were most cluster RCTs. The observational studies were of mixed quality. We were only able to meta-analyse case-control data. We searched for any interventions to prevent viral transmission of respiratory viruses (isolation, quarantine, social distancing, barriers, personal protection and hygiene). Study design included RCTs, cohort studies, casecontrol studies, cross-over studies, before-after, and time series studies. Data collection and analysis: We scanned the titles, abstracts and full text articles using a standardised form to assess eligibility. RCTs were assessed according to randomisation method, allocation generation, concealment, blinding, and follow up. Non-RCTs were assessed for the presence of potential confounders and classified as low, medium, and high risk of bias.

MAIN RESULTS: The highest quality cluster RCTs suggest respiratory virus spread can be prevented by hygienic measures around younger children. Additional benefit from reduced transmission from children to other household members is broadly supported in results of other study designs, where the potential for confounding is greater. The six case-control studies suggested that implementing barriers to transmission, isolation, and hygienic measures are effective at containing respiratory virus epidemics. We found limited evidence that the more uncomfortable and expensive N95 masks were superior to simple surgical masks. The incremental effect of adding virucidals or antiseptics to normal handwashing to decrease respiratory disease remains uncertain. The lack of proper evaluation of globalmeasures such as screening at entry ports and social distancing prevent firm conclusions about these measures.

AUTHORS’ CONCLUSIONS: Many simple and probably low-cost interventions would be useful for reducing the transmission of epidemic respiratory viruses. Routine long-term implementation of some of the measures assessed might be difficult without the threat of a looming epidemic.

## 2. Vaccines for preventing influenza in the elderly^2^

ABSTRACT

BACKGROUND: Influenza vaccination of elderly individuals is recommended worldwide and has been targeted toward the elderly and those at serious risk of complications.

OBJECTIVES: Our aim was to review the evidence of efficacy, effectiveness and safety of influenza vaccines in individuals aged 65 years or older.

SEARCH STRATEGY: We searched the Cochrane Central Register of Controlled Trials (CENTRAL), which contains the Cochrane Acute Respiratory Infection (ARI) Group’s specialized register, the Cochrane Database of Systematic Reviews, and the Database of Abstracts of Reviews of Effectiveness, (2006, issue 1); MEDLINE (January 1966 to March Week 3 2006); EMBASE (Dialog 1974 to 1979; SilverPlatter 1980 to December 2005); Biological Abstracts (SilverPlatter 1969 to December 2004); and Science Citation Index (Web of Science 1974 to December 2004).

SELECTION CRITERIA: We considered randomised, quasi-randomised, cohort and case-control studies assessing efficacy against influenza (laboratory-confirmed cases) or effectiveness against influenza-like illness (ILI) or safety. Any influenza vaccine given independently, in any dose, preparation or time schedule, compared with placebo or with no intervention was considered.

DATA COLLECTION AND ANALYSIS: We grouped reports first according to the setting of the study (community or long-term care facilities) and then by level of viral circulation and vaccine matching. We further stratified by co-administration of pneumococcal polysaccharide vaccine (PPV) and by different types of influenza vaccines. We analysed the following outcomes: influenza, influenza-like illness, hospital admissions, complications and deaths.

MAIN RESULTS: Sixty-four studies were included in the efficacy/effectiveness assessment, resulting in 96 data sets. In homes for elderly individuals (with good vaccine match and high viral circulation) the effectiveness of vaccines against ILI was 23% (6% to 36%) and non-significant against influenza (risk relative, RR 1.04: 95% confidence interval, CI 0.43 to 2.51). We found no correlation between vaccine coverage and ILI attack rate. Well matched vaccines prevented pneumonia (VE 46%; 30% to 58%), hospital admission (VE 45%; 16% to 64%) and deaths from influenza or pneumonia (VE 42%, 17% to 59%). In elderly individuals living in the community, vaccines were not significantly effective against influenza (RR 0.19; 95% CI 0.02 to 2.01), ILI (RR 1.05: 95% CI 0.58 to 1.89), or pneumonia (RR 0.88; 95% CI 0.64 to 1.20). Well matched vaccines prevented hospital admission for influenza and pneumonia (VE 26%; 12% to 38%) and all-cause mortality (VE 42%; 24% to 55%). After adjustment for confounders, vaccine performance was improved for admissions to hospital for influenza or pneumonia (VE* 27%; 21% to 33%), respiratory diseases (VE* 22%; 15% to 28%) and cardiac disease (VE* 24%; 18% to 30%); and for all-cause mortality (VE* 47%; 39% to 54%). The public health safety profiles of the vaccines appear to be acceptable.

AUTHORS’ CONCLUSIONS: In long-term care facilities, where vaccination is most effective against complications, the aims of the vaccination campaign are fulfilled, at least in part. However, according to reliable evidence the usefulness of vaccines in the community is modest. The apparent high effectiveness of the vaccines in preventing death from all causes may reflect a baseline imbalance in health status and other systematic differences in the two groups of participants.
